# Genomic surveillance for hypervirulence and multi-drug resistance in invasive *Klebsiella pneumoniae* from South and Southeast Asia

**DOI:** 10.1186/s13073-019-0706-y

**Published:** 2020-01-16

**Authors:** Kelly L. Wyres, To N. T. Nguyen, Margaret M. C. Lam, Louise M. Judd, Nguyen van Vinh Chau, David A. B. Dance, Margaret Ip, Abhilasha Karkey, Clare L. Ling, Thyl Miliya, Paul N. Newton, Nguyen Phu Huong Lan, Amphone Sengduangphachanh, Paul Turner, Balaji Veeraraghavan, Phat Voong Vinh, Manivanh Vongsouvath, Nicholas R. Thomson, Stephen Baker, Kathryn E. Holt

**Affiliations:** 1 Department of Infectious Diseases, Central Clinical School, Monash University, Melbourne, Victoria 3004 Australia; 2 Hospital of Tropical Diseases, Oxford University Clinical Research Unit, Ho Chi Minh City, Vietnam; 3 The Hospital of Tropical Diseases, Ho Chi Minh City, Vietnam; 4 Lao-Oxford-Mahosot Hospital-Wellcome Trust Research Unit, Microbiology Laboratory, Mahosot Hospital, Vientiane, Lao People’s Democratic Republic; 5 Centre for Tropical Medicine and Global Health, University of Oxford, Oxford, UK; 6 London School of Hygiene and Tropical Medicine, London, UK; 7 Department of Microbiology, The Chinese University of Hong Kong, Hong Kong Special Administrative Region, China; 8 Patan Academy of Health Sciences, Oxford University Clinical Research Unit, Kathmandu, Nepal; 9 Shoklo Malaria Research Unit, Mahidol-Oxford Tropical Medicine Research Unit, Faculty of Tropical Medicine, Mahidol University, Mae Sot, 63110 Thailand; 10 Cambodia Oxford Medical Research Unit, Angkor Hospital for Children, Siem Reap, Cambodia; 11 Department of Clinical Microbiology, Christian Medical College, Vellore, Tamil Nadu India; 12 Wellcome Trust Sanger Institute, Hinxton, Cambridge, UK; 13 Cambridge Institute of Therapeutic Immunology & Infectious Disease (CITIID) Department of Medicine, University of Cambridge, Cambridge Biomedical Campus, Cambridge, CB2 0AW UK

**Keywords:** *Klebsiella pneumoniae*, Bloodstream infection, MDR, Capsule types, Hypervirulent, Genomic surveillance

## Abstract

**Background:**

*Klebsiella pneumoniae* is a leading cause of bloodstream infection (BSI). Strains producing extended-spectrum beta-lactamases (ESBLs) or carbapenemases are considered global priority pathogens for which new treatment and prevention strategies are urgently required, due to severely limited therapeutic options. South and Southeast Asia are major hubs for antimicrobial-resistant (AMR) *K. pneumoniae* and also for the characteristically antimicrobial-sensitive, community-acquired “hypervirulent” strains. The emergence of hypervirulent AMR strains and lack of data on exopolysaccharide diversity pose a challenge for *K. pneumoniae* BSI control strategies worldwide.

**Methods:**

We conducted a retrospective genomic epidemiology study of 365 BSI *K. pneumoniae* from seven major healthcare facilities across South and Southeast Asia, extracting clinically relevant information (AMR, virulence, K and O antigen loci) using *Kleborate*, a *K. pneumoniae*-specific genomic typing tool.

**Results:**

*K. pneumoniae* BSI isolates were highly diverse, comprising 120 multi-locus sequence types (STs) and 63 K-loci. ESBL and carbapenemase gene frequencies were 47% and 17%, respectively. The aerobactin synthesis locus (*iuc*), associated with hypervirulence, was detected in 28% of isolates. Importantly, 7% of isolates harboured *iuc* plus ESBL and/or carbapenemase genes. The latter represent genotypic AMR-virulence convergence, which is generally considered a rare phenomenon but was particularly common among South Asian BSI (17%). Of greatest concern, we identified seven novel plasmids carrying both *iuc* and AMR genes, raising the prospect of co-transfer of these phenotypes among *K. pneumoniae*.

**Conclusions:**

*K. pneumoniae* BSI in South and Southeast Asia are caused by different STs from those predominating in other regions, and with higher frequency of acquired virulence determinants. *K. pneumoniae* carrying both *iuc* and AMR genes were also detected at higher rates than have been reported elsewhere. The study demonstrates how genomics-based surveillance—reporting full molecular profiles including STs, AMR, virulence and serotype locus information—can help standardise comparisons between sites and identify regional differences in pathogen populations.

**Electronic supplementary material:**

The online version of this article (10.1186/s13073-019-0706-y) contains supplementary material, which is available to authorized users.

## Background

*Klebsiella pneumoniae* is regarded globally by the World Health Organization (WHO) as a priority antimicrobial-resistant (AMR) pathogen requiring new control strategies [1]. These include rapid identification and containment of high-risk AMR clones such as the carbapenemase-producing (CP) variants, augmented with vaccines, bacteriophages, or immunotherapies that target surface antigens. However, *K. pneumoniae* is highly diverse, hindering the development of such strategies and our ability to study its molecular epidemiology in a relevant time-frame.

This diverse bacterial species is generally associated with a range of differing community and healthcare-associated infections, but can be particularly problematic when the organisms gain access to sterile sites such as the cerebrospinal fluid and the bloodstream. Such infections are often characterised by rapid onset and multi-drug resistance (MDR), including resistance to third-generation cephalosporins and/or carbapenems. Antimicrobials are the primary treatment strategy but options can be severely limited by AMR, particularly for third-generation cephalosporin- and carbapenem-resistant strains causing invasive infections [2]. Concomitant with this are elevated mortality rates and treatment costs [3].

*K. pneumoniae* is among the most common cause of bloodstream infections (BSI) in South (S) and Southeast (SE) Asia [4–6] where it is associated with a high mortality rate [4, 7]. Available data suggest a heterogenous landscape in terms of drug resistance; for example, CP strains are rare in SE Asia (< 1–4% [5, 8, 9]) but common in S Asia (28–70% [10, 11]) and the prevalence of extended-spectrum beta-lactamase (ESBL, confers resistance to the third-generation cephalosporins) producing organisms varies from 12 to 79% in these regions [4–6, 8, 10]. Studies investigating ESBL and CP variants in S/SE Asia implicate CTX-M-15 as the most common ESBL type [12, 13], while NDM and OXA-48-like enzymes are the most commonly described carbapenemases [11, 14–16]. However, there is currently only limited information about the underlying population structure of these organisms in terms of multi-locus sequence types (STs), or genomically defined phylogenetic lineages.

Searching PubMed for reports of multi-locus sequence typing (MLST) or whole-genome sequence analysis of *K. pneumoniae* BSI (see “Methods” for search terms) yielded just nine studies reporting ST information for India, Nepal, Vietnam, Thailand, Laos, Cambodia and/or Hong Kong (the foci of this work). The majority of these (six of nine) were case reports or genome announcements from India, while an additional study from India described a screen of Enterobacteriaceae isolated from neonatal sepsis, including 12 *K. pneumoniae* [17–24]. Together, these works from India report ST information for a single ESBL-producing strain (ST2318) and 24 CP strains; five each of ST231 and ST347; two each of ST29, ST147, ST1224 and ST2558; and one each of ST11, ST43 and ST101. Carbapenemase genes were reported for 21 of the CP strains and included 11 *bla*_NDM-1_, 8 *bla*_OXA-48-like_, one each of *bla*_NDM-7_ and *bla*_KPC_. A single report from Nepal described two neonatal sepsis outbreaks [7], the first caused by ST15 carrying *bla*_NDM-1_ and *bla*_CTX-M-15_, and the second by ST1559 carrying *bla*_CTX-M-15_ without any carbapenemases. The final report described *K. pneumoniae* BSI isolates from Hong Kong that produced DHA enzymes (resulting in third-generation cephalosporin resistance). Among the genotyped isolates were four ST11, and one each of ST17, ST23 and ST39 [25].

It is clear from these reports that several well-known globally distributed CP/ESBL-associated STs are present in S/SE Asia, e.g. STs 14, 15, 17, 29, 101, 147 and 231, a proposition that is further supported by studies exploring *K. pneumoniae* isolates from a broader range of clinical specimen types [12, 13, 15, 26, 27]. However, there remains a clear lack of systematic studies with which to fully understand the CP, ESBL and broader population genotypes of *K. pneumoniae* causing BSI in these regions.

Capsule- or lipopolysaccharide- (LPS) targeted immunisation against *K. pneumoniae* has been proposed as an alternative strategy for prevention or therapy of MDR BSI. Hence, capsular serotype (K-type) and LPS (O-type) profiles among BSI isolates are also of interest. More than 130 capsular serotypes have been predicted on the basis of genome data [28]. Among the studies identified through the formal search described above, two did not report K-types and three included only a limited assessment, i.e. PCR screening for a small number of types that are associated with enhanced virulence (K1 and K2 [17, 24], or K1, K2, K5, K20, K54 and K57 [23]). Among the remaining three studies, two reported single K17 and K64 CP isolates in India [19, 20], while the Nepalese outbreak study reported the KL14 capsule locus, encoding capsule type K14 (ST1559) and a novel locus that was later defined as KL112 (ST15) [7]. None of these studies reported O-types. We searched PubMed for additional studies reporting K- and O-types of *K. pneumoniae* BSI in our regions of interest (search terms in “Methods”), revealing one additional report describing 47 isolates (including 28 BSI isolates) from a suspected neonatal intensive care unit outbreak in Madras, India [29]: Thirty-eight isolates were shown to be closely related by pulsed-field gel electrophoresis (MLST not reported), all of which expressed the same novel K and O serotype. Among the remaining nine unrelated isolates, three K2:O1 were identified in addition to one each of K23:O1, K54:O1, K62:O1, K62:O2, K-non-typeable:O1 and K2:O-novel.

The capsule, alongside the LPS, type I and type III fimbriae, and the enterobactin siderophore, are common to all *K. pneumoniae* strains and are required for pathogenesis. A number of accessory virulence determinants (including RmpA/RmpA2, which upregulate capsule expression; the colibactin genotoxin; and the yersiniabactin, aerobactin and salmochelin siderophores that promote systemic survival and dissemination [30–34]) are overrepresented among isolates causing invasive disease in human population studies [35–37]. Each has also been shown to enhance virulence in murine infection models through comparison of isogenic knockout mutant strains [31–34]. *K. pneumoniae* expressing several of these determinants are associated with severe community-acquired invasive disease, often manifesting as liver abscess. These are classified as “hypervirulent” infections; they occur globally but are most commonly reported in SE Asia [38]. They are predominantly associated with clones ST23, ST86 and ST65 [39, 40], which typically express the K1/K2 capsules. The dominance of a small number of clones combined with strong linkage between the *rmpA/A2*, aerobactin and salmochelin synthesis loci (which are co-located on virulence plasmids [41, 42]) has created difficulties for understanding the relative importance of these key virulence factors. However, recent studies implicate aerobactin as the most important siderophore and a key biomarker for hypervirulence alongside the *peg-344* transporter gene (sometimes misannotated as *pagO*, in strong genetic linkage with aerobactin due to being located nearby on the same virulence plasmid) [37, 43, 44]. Notably, despite its apparent relevance as a hypervirulence biomarker, *peg-344* isogenic knockout mutants did not show reduced virulence in either of two independent murine models of hypervirulent disease [44, 45].

To date the majority of hypervirulent *K. pneumoniae* have remained susceptible to antimicrobials [38, 46] (except ampicillin). However, the last few years have seen increasing reports of “convergent” *K. pneumoniae* that are both hypervirulent (defined as strains carrying the *iuc* aerobactin locus) and MDR ESBL/CP either due to acquisition of an MDR plasmid by a hypervirulent strain [47–51] or by acquisition of a virulence plasmid by an MDR strain [49, 52–56]. The majority of these reports represent sporadic isolations. However in 2017, it was reported that an outbreak of ventilator-associated pneumonia in a Chinese hospital, resulting in five deaths, was caused by a CP ST11 strain that had acquired a virulence plasmid harbouring the *iuc* locus, plus *rmpA2* (without the salmochelin locus (*iro*), *rmpA* or *peg-344*) [52]. Notably, the outbreak strain showed enhanced virulence in the *Galleria mellonella* infection model compared to a wild-type ST11 lacking the virulence plasmid and a plasmid cured variant [52], further supporting the importance of the *iuc* locus. Subsequent retrospective investigations revealed that similar CP ST11 *iuc +* strains had already been silently spreading within China prior to the initial report [52, 57].

Given the high burden of both MDR and hypervirulent *K. pneumoniae* infections, S/SE Asia may represent a hub for MDR-virulence convergence, with the potential for outbreaks of severe disease with extremely limited treatment options such as that reported in China [52]. However, so far no studies have provided a complete molecular epidemiological picture (STs, serotypes, AMR *and* hypervirulence determinants) of *K. pneumoniae* BSI agents in S and SE Asia, leaving a gap in our knowledge about the local pathogen population and the prevalence and/or diversity of convergent strains. Here we present a genomic epidemiology study of BSI *K. pneumoniae* from seven major healthcare facilities across S/SE Asia, leveraging a recently established genomic framework that incorporates rapid genotyping of clinically important features (ST, AMR, hypervirulence, and capsule and lipopolysaccharide synthesis loci). The data are highly relevant to the design of *K. pneumoniae* control strategies, revealing a diverse population with high rates of AMR and virulence loci, and high prevalence of convergent strains.

## Methods

### Literature review

To identify studies reporting molecular data on *K. pneumoniae* BSI in our regions of interest, we searched PubMed with no language restrictions for reports that contained the terms “(Klebsiella)” and “((India*) OR (Nepal*) OR (Viet*) OR (Hong Kong) OR (Cambodia) OR (Lao*) OR (Thai*) OR (Asia))” and “(blood*) OR (sepsis) OR (bacteraemia) OR (bacteremia)” and “(MLST) OR (sequence type) OR (multi-locus sequence typ*) OR (genom*)”. To check for other studies reporting *K. pneumoniae* serotypes in the region, we searched for “(*Klebsiella)*” and “((India*) OR (Nepal*) OR (Viet*) OR (Hong Kong) OR (Cambodia) OR (Lao*) OR (Thai*) OR (Asia))” and “(blood*) OR (sepsis) OR (bacteraemia) OR (bacteremia)” and “(serotyp*) OR (capsul*) OR (K-type) OR (O-type) OR (LPS) OR (lipopolysaccharide)”.

### Setting

The following tertiary care hospitals were included: Patan Hospital, Kathmandu, Nepal; Christian Medical College Hospital, Vellore, India; Angkor Hospital for Children, Siem Reap, Cambodia; Mahosot Hospital, Vientiane, Lao PDR; The Hospital of Tropical Diseases, Ho Chi Minh City, Vietnam; Prince of Wales Hospital, Hong Kong. Isolates were also provided from healthcare inpatient departments serviced by the Shoklo Malaria Research Unit, Mae Sot, Thailand.

### Bacterial isolates and culture

**Fig. 1:**
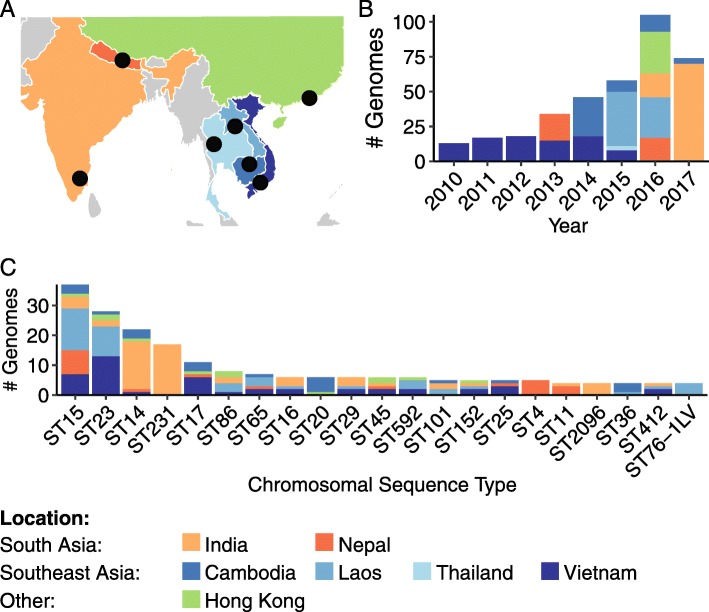
*Klebsiella pneumoniae* BSI isolates included in this study. **a** Collection sites and countries of origin for all *Klebsiella pneumoniae* complex isolates for which genome data were available. **b** Years of collection coloured by country of origin as in panel **a**. **c** Chromosomal multi-locus sequence types (STs) of *Klebsiella pneumoniae* sensu stricto isolates (only STs accounting for > 1% isolates are shown, coloured by country of origin as in panel **a**)

*K. pneumoniae* isolates obtained from blood cultures derived from hospital- and/or community-associated BSI, following routine diagnostic protocols in each hospital laboratory and identified using biochemical testing (typically API-20E, bioMerieux), were included in the study. Isolates available for sequencing represented the following fractions of *K. pneumoniae* BSI collected at each site in participating years: India, 10%; Hong Kong, 17%; Vietnam, 20%; other sites, > 90% (Fig. 1). All sequenced isolates represent unique patient infection episodes.

### DNA extractions, library preparation and sequencing

All isolates were cultured in LB broth at 37 °C overnight before DNA extraction. Multiplexed Nextera XT libraries were sequenced on Illumina platforms, generating 150 bp paired-end reads. Eight isolates were selected for additional long-read sequencing using the Nanopore MinION R9 device as previously described [58].

### Genome assembly and genotyping

Illumina adapter sequences were removed and reads were quality trimmed using TrimGalore v0·4·4 (https://github.com/FelixKrueger/TrimGalore). Subsequently, draft de novo assemblies were generated using SPAdes v3·10·1 [59] optimised with Unicycler v0·4·7 [60]. We excluded from further analysis nine low-quality genome assemblies outside the expected size range (5–6·5 Mbp). Chromosomal MLST, virulence locus (*ybt*, *iro*, *iuc*, *rmpA*, *rmpA2*), and acquired resistance genes (excluding the core ampicillin resistance gene *bla*_SHV_ and *oqxAB* efflux genes) were typed using *Kleborate* v0·3·0 (https://github.com/katholt/Kleborate). The *peg-344* gene was identified using BLASTn search of the genome assemblies (query sequence accession BAH65947.1, ≥ 90% coverage and identity). Capsule (K) and lipopolysaccharide (O) loci were identified using *Kaptive* [28, 61]. Note that the KL1-KL77 loci are associated with the serologically defined capsule types K1-K77, respectively [62]. Serological types are yet to be resolved for the remaining loci (KL101-KL161), which were defined previously on the basis of gene content such that they are predicted to encode distinct capsule types [28, 63]. Novel K-loci were manually extracted using Bandage [64] and annotated using Prokka v1.13.3 [65]. Where *Kaptive* was unable to confidently identify a K-locus due to fragmented genome assemblies (*n* = 129), K-loci were predicted from *wzi* alleles as described previously [66]. *Wzi* allelic typing was considered the gold-standard capsule genotyping method prior to the introduction of full-locus-based typing. However, when genome data are available, the latter approach is preferred because the K-locus is subject to chromosomal recombinations that can lead to a breakdown of associations between individual *wzi* allelic variants and K-locus types [28]. As a result, a single *wzi* allele may be associated with multiple distinct K-loci. Such alleles were identified among 29 genomes in this study, resulting in ambiguous K-locus calls that were excluded from further analyses. An additional 11 and one genomes harboured novel or missing *wzi* alleles, respectively for which no K-locus predictions were possible.

*Catpac* [67] was used to calculate pairwise nucleotide differences between genomes after reassembly with SKESA v2.2.1 [68] (a highly conservative assembly approach, which reduces the likelihood of false-positive nucleotide differences). These data were used to identify clusters of isolates for which the genomes differed by ≤ 100 and ≤ 25 single nucleotide variants (SNVs). Isolates from the same facility and separated by ≤ 25 SNVs can be considered putative nosocomial transmission clusters [67, 69, 70]; distances ≤ 100 SNVs also imply descent from a recent common ancestor, but are more consistent with community or between-facility transmission, or regional spread between countries [70].

Hybrid Illumina-Nanopore assemblies were generated using Unicycler v0·4·7 as described previously [58]. Assemblies were annotated using Prokka v1·13·3 and *iuc +* plasmid annotations were manually curated before depositing in GenBank (accessions listed in Table 4). Plasmid replicon types were identified using the PlasmidFinder database v2·0 [71]. Isolate information, genotypes and genome data accessions are provided in Additional file 1: Table S1. Genome assemblies, novel plasmid and K-locus sequences are also available in Figshare [72]: 10.26180/5c67982956721.

### Statistical analyses

Statistical analyses were performed in R v3·3·3 [73], and data were visualised using ggplot2 v2·2·1 [74]. Given the disparity in sampling frames and small subgroup sample sizes, it was not appropriate to test trends at a country or year level. Statistical tests for regional differences in genetic features between *K. pneumoniae* populations (Table 1) were calculated using the subset of *K. pneumoniae* collected in overlapping 2-year periods: S Asia region (Nepal and India; 2016–2017; *n* = 102) vs SE Asia region (Cambodia, Laos, Thailand, Vietnam; 2015–2016, *n* = 100).

**Table 1:** Comparison of key features of *Klebsiella* genomes from South and Southeast Asian sites

	S Asia	SE Asia	*p* value (adj)	OR	LCI	UCI	
*Species assignment*	*102*	*100*					
*K. pneumoniae*	100 (98%)	87 (87%)	0.011	0.13	0.01	0.62	*
*K. variicola*	1 (1%)	4 (4%)	0.838	4.18	0.40	209.04	
*K. quasipneumoniae* ssp. *quasipneumoniae*	1 (1%)	0 (0%)	na	na	na	na	
*K. quasipneumoniae* ssp. *similipneumoniae*	0 (0%)	9 (9%)	na	na	na	na	
Features of *K. pneumoniae* sensu stricto							
*Sequence type assignment*	*100*	*87*					
ST15	11 (11%)	15 (17%)	1.158	1.68	0.67	4.32	
ST14	16 (16%)	2 (2%)	0.008	0.12	0.01	0.56	**
ST23	2 (2%)	12 (14%)	0.015	7.76	1.65	73.39	*
ST231	17 (17%)	0 (0%)	5.14 × 10^−5^	0.00	0.0	0.24	**
*Capsular serotype prediction*	*79*	*87*					
KL1	2 (2.5%)	13 (15%)	0.012	6.70	1.44	63.22	*
KL2	4 (5.1%)	11 (13%)	0.216	2.70	0.76	12.15	
*Antimicrobial resistance prediction*	*100*	*87*					
Multi-drug resistant (≥ 3 acquired classes)	81 (81%)	44 (51%)	1.06 × 10^−4^	0.24	0.12	0.48	**
Aminoglycosides	76 (76%)	41 (47%)	0.001	0.28	0.14	0.55	**
Fluoroquinolones	80 (80%)	39 (45%)	7.13 × 10^−6^	0.21	0.10	0.41	**
Phenicols	27 (27%)	25 (29%)	7.833	1.09	0.55	2.17	
Sulfonamides	63 (63%)	42 (48%)	0.493	0.55	0.29	1.02	
Tetracyclines	24 (24%)	39 (45%)	0.029	2.56	1.32	5.05	*
Trimethoprim	62 (62%)	38 (44%)	0.118	0.48	0.25	0.89	
3rd-generation cephalosporins (ESBL)	60 (60%)	41 (47%)	0.949	0.60	0.32	1.11	
Carbapenems	47 (47%)	1 (1%)	7.13 × 10^−13^	0.01	0.0	0.08	**
*Virulence prediction*	*100*	*87*					
Yersiniabactin (*ybt*)	65 (65%)	46 (53%)	0.205	0.61	0.32	1.13	
Aerobactin (*iuc*)	27 (27%)	32 (37%)	0.319	1.57	0.81	3.07	
*Iuc* lineages	*100*	*87*					
*iuc 1*	13 (13%)	27 (31%)	0.012	2.99	1.36	6.86	*
*iuc 3*	0 (0%)	5 (6%)	0.061	na	1.08	inf	
*iuc 5*	12 (12%)	0 (0%)	0.001	0.00	0.0	0.8	**
*AMR and virulence convergence*	*100*	*87*					
ESBL and *ybt*	45 (45%)	26 (30%)	0.144	0.52	0.27	0.99	
ESBL and *iuc*	16 (16%)	1 (1%)	0.001	0.06	0.0	0.41	**
CP and *ybt*	37 (37%)	1 (1%)	1.79 × 10^− 10^	0.02	0.0	0.13	**
CP and *iuc*	13 (13%)	0 (0%)	na	na	0.0	0.34	

*South (S) Asia is represented by the two sites in India and Nepal, isolated 2016–2017; Southeast (SE) Asia is represented by the sites in Cambodia, Laos, Thailand and Vietnam, isolated 2015–2016. *p* values were calculated using Fisher’s exact test for differences in the prevalence of each feature (in rows) for the S vs SE Asia samples and are adjusted using Bonferroni correction for the number of tests within each group of comparisons (as labelled in italics; sample size for each are also in italics). *na* test not applicable (one or more values equal to zero). *Inf* infinity, *OR* odds ratio, LCI and UCI, lower and upper bounds for 95% confidence interval, respectively. *p < 0·05, **p < 0·01

## Results

The genomes of 393 presumptive *K. pneumoniae* BSI from seven countries across S/SE Asia were sequenced (Fig. 1a, Additional file 1: Table S1). Twenty-eight genomes were identified as Enterobacteriaceae species outside of the *K. pneumoniae* species complex and were excluded. Among the remaining 365 isolates, the majority (*n* = 331, 91%) of organisms were confirmed to be *K. pneumoniae*. Among the regional comparator samples, these accounted for a higher proportion of genomes from S than SE Asia (98% vs 87%, *p* = 0·011; Table 1). The remaining isolates (Additional file 2: Table S2) were *K. quasipneumoniae* subsp*. similipneumoniae* (*n* = 20, 5·5%), *K. variicola* (*n* = 9, 2·5%) and *K. quasipneumoniae* subsp. *quasipneumoniae* (*n* = 5, 1·4%), which are indistinguishable from *K. pneumoniae* by microbiological methods [35, 67]. Unfortunately, we did not have access to patient clinical data and hence were not able to assess the likelihood that individual BSI cases were hospital- or community-acquired, or to explore patient co-morbidities, which we expect to be quite diverse.

The 331 *K. pneumoniae* were highly diverse and comprised 120 individual STs (Simpson’s diversity index = 0·97), the majority (61%) of which were represented by a single isolate. Nevertheless, we observed four common STs that each accounted for > 5% of the sequenced organisms: ST15 (*n* = 37, 11%), ST23 (*n* = 28, 8·5%), ST14 (*n* = 22, 6·6%) and ST231 (*n* = 17, 5·1%; Fig. 1c). Among these, only ST15 was common across all sites, whereas ST23 was significantly associated with SE Asia (*p* = 0·015) and ST14 and ST231 were both significantly associated with S Asia (*p* < 0·01; Table 1).

### Predicted capsular (K) and O antigen serotypes

**Fig. 2:**
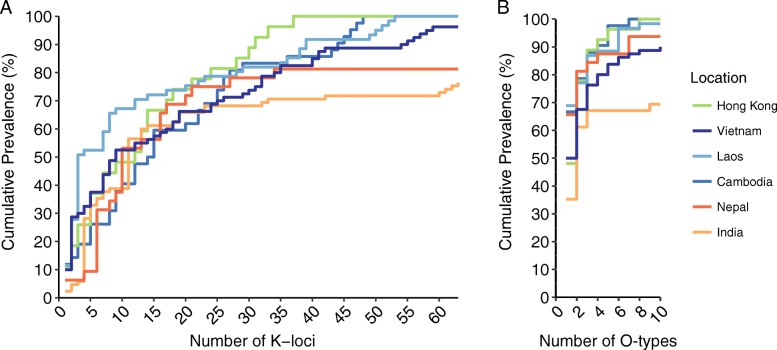
Cumulative K and O locus prevalence by location. **a** Cumulative prevalence of K-loci ordered by mean prevalence across all locations (highest to lowest, see Additional file 2: Table S3). **b** Cumulative prevalence of predicted O-types ordered by mean prevalence across all locations (highest to lowest, see Additional file 2: Table S4). Note that the Thai sampling site is excluded due to small sample size (*n* = 4)

In this collection of invasive *K. pneumoniae* from BSI, we detected 63 different K-loci including four novel loci designated KL162–165 (GenBank accessions: MK593451- MK593454, Simpson’s diversity index = 0·95), the majority (67%) of these K-loci were found in ≤ 3 *K. pneumoniae* isolates each (Additional file 2: Table S3). The most common K-loci were KL1 (*n* = 31; including 28 ST23), KL2 (*n* = 27; numerous STs including ST14, ST25, ST65 and ST86), KL51 (*n* = 23; including 17 ST231) and KL24 (*n* = 20; including 17 ST15), together accounting for 28% of *K. pneumoniae* BSI (Fig. 2). Saliently, while ST23 and ST231 were each associated with only a single K-locus (KL1 and KL51, respectively), the two other most common STs were each associated with multiple K-loci (ST15: KL24 = 17, KL112 = 9, KL10 = 3, KL62 = 2, KL19 = 2; ST14: KL64 = 7, KL2 = 6, KL157 = 1), as has been previously observed for ST258 [61]. Ten of the 12 previously described O antigen encoding loci were also detected (Simpson’s diversity index = 0·80). Loci predicted to encode serotypes O1 and O2 were the most common, together accounting for 71% of *K. pneumoniae* BSI (Fig. 2, Additional file 2: Table S4). Notably, the O3b locus, which is considered to be rare [63], was detected here at 8% prevalence across sites (mean 7% per site, range 0–11%).

### Acquired antimicrobial resistance determinants

**Fig. 3:**
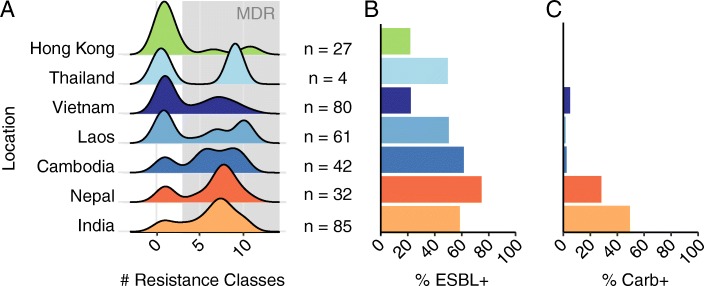
Prevalence of antimicrobial resistance determinants among *K. pneumoniae* sensu stricto isolates. **a** Density plots showing the distributions of number of drug classes for which acquired resistance determinants were detected in each genome (*n* = total genomes by location). Grey shading indicates multi-drug resistance (≥ 3 resistance classes). **b** Proportion of genomes for which extended-spectrum beta-lactamase genes were detected (% ESBL+). **c** Proportion of genomes for which carbapenemase genes were detected (% Carb+)

Acquired AMR determinants were detected in 91% of *K. pneumoniae* genomes. The number of antimicrobial classes to which each isolate was predicted to be resistant showed a bimodal distribution (Fig. 3a), with the majority of *K. pneumoniae* being MDR (acquired AMR genes conferring resistance to ≥ 3 drug classes; 63%) or possessing no acquired AMR genes (9%). The prevalence of MDR differed between sampling locations and ranged from 22% in Hong Kong to 85% in India. MDR was significantly more prevalent among S Asian isolates than SE Asian isolates (81% vs 51%, *p* = 0·0001; Table 1). Consistently, the S Asian organisms had a significantly higher prevalence of fluoroquinolone, aminoglycoside and carbapenem resistance determinants than the SE Asian organisms (*p* < 0·001 for each class; Table 1).

The overall prevalence of ESBL genes among the *K. pneumoniae* isolates was 47% (*n* = 157), but varied between study sites (22–75%; Fig. 3b). The majority of ESBL *K. pneumoniae* (90%) were predicted to be resistant to a further ≥ 3 alternative antimicrobial classes (median 7 classes). The most common ESBL genes were *bla*_CTX-M-15_ (*n* = 120/157, 76%), *bla*_CTX-M-27_ (*n* = 14/157, 9%) and *bla*_CTX-M-14_ (*n* = 13/157, 8%); these were detected in diverse chromosomal STs (Table 2, Additional file 1: Table S1). Twelve additional putative ESBL genes were also detected in 1–7 genomes each (Additional file 1: Table S1).

**Table 2:** Notable AMR STs by location

Location	Total ESBL (%)	Total CP (%)	Notable clone/s	*N*	N ESBL (% of ESBL/site)	ESBL genes (*N*)	*N* CP (% of CP/site)	CP genes (*N*)
Hong Kong	5 (19%)	0	–	–	–	–	–	–
Vietnam	20 (25%)	4 (5%)	* ST15	7	4 (20%)	CTX-M-15 (3) CTX-M-14 (2)	2 (50%)	NDM-1 (1)
Thailand	2 (50%)	0	–	–	–	–	–	–
Laos	32 (53%)	1 (2%)	* ST15	14	14 (44%)	CTX-M-15 (14)	0	–
			ST76-1LV	4	4 (13%)	CTX-M-15 (4)	0	–
Cambodia	27 (64%)	1 (2%)	ST20	5	5 (19%)	CTX-M-27 (3) CTX-M-14 (2) CTX-M-15 (1)	0	–
			* ST15	3	3 (11%)	CTX-M-15 (3) CTX-M-27 (1)	0	–
			ST36	3	3 (11%)	CTX-M-15 (2)	0	–
			* ST15	8	8 (33%)	CTX-M-15 (8)	6 (67%)	NDM-1 (5)
Nepal	24 (75%)	9 (28%)	ST4	5	5 (21%)	CTX-M-15 (5)	0	–
			ST11	3	3 (13%)	SFO-1 (3)	3 (33%)	OXA-232 (3)
India	52 (61%)	42 (49%)	ST231	17	13 (25%)	CTX-M-15 (13)	13 (31%)	OXA-232 (13)
			ST14	16	7 (13%)	CTX-M-15 (7)	11 (26%)	OXA-232 (10)
			* ST15	4	3 (6%)	CTX-M-15 (3)	0	–
			ST16	3	3 (6%)	CTX-M-15 (3)	2 (5%)	NDM-1 (1)
			ST29	3	3 (6%)	CTX-M-15 (3)	0	–
			ST395	3	1 (25%)	CTX-M-15 (1)	3 (7%)	OXA-232 (3)

Total ESBL and Total CP: total number of genomes carrying ESBL and carbapenemase genes per site, respectively

Notable clones: clones with ≥ 3 ESBL and/or carbapenemase gene-positive genomes at any single site. *ST15 is the only clone meeting these criteria at > 1 site

*N*: total number of genomes of each notable clone at each site (includes all genomes assigned to the ST regardless of predicted AMR)

*N* ESBL: number of genomes of each notable clone with at least one ESBL and no carbapenemase genes per site

ESBL genes: details of the ESBL genes detected in each notable clone at each site

*N* CP: number of genomes of each notable clone with at least one carbapenemase gene per site

CP genes: details of the carbapenemase genes detected in each notable clone at each site

The overall prevalence of carbapenemase genes was 17% (*n* = 57), again varying widely between sites (0–50%; Fig. 3c). All isolates with a carbapenemase gene were also MDR, with predicted resistance to a median of six drug classes. The most common carbapenemases were the OXA-48-like *bla*_OXA-232_ (*n* = 36/57; 63%) and the metallo-beta-lactamase *bla*_NDM-1_ (*n* = 18/57; 32%, including four genomes with *bla*_OXA-232_); again, these were each detected in a diverse set of STs (Table 2, Additional file 1: Table S1). Five other carbapenemase genes were detected in 1–4 genomes each (Additional file 1: Table S1); *bla*_KPC_ was not detected. Details of ESBL/CP *K. pneumoniae* STs identified at each site, including their specific enzymes, are shown in Table 2. Notably ST15 carrying *bla*_CTX-M-15_ were identified at all sites except Thailand, and occasionally also harboured the carbapenemases *bla*_NDM-1_ or *bla*_OXA-232_.

### Acquired virulence determinants

**Fig. 4:**
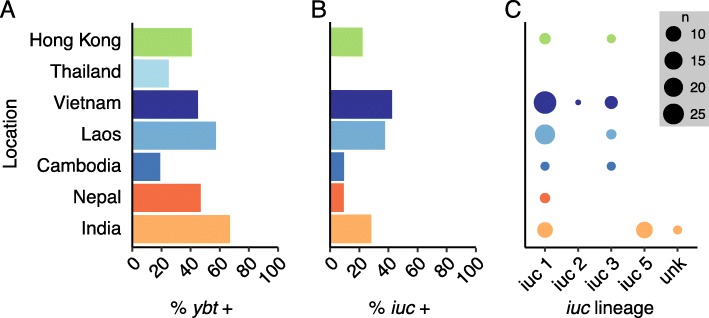
Prevalence of key virulence determinants among *K. pneumoniae* sensu stricto isolates. **a** Proportion of genomes for which the yersiniabactin locus was detected (% *ybt*+) by location. **b** Proportion of genomes for which the aerobactin locus was detected (% *iuc*+) by location. **c** *iuc* lineages by location. Points are scaled by the number of genomes as per legend. Unk; unknown

We identified genes linked to invasive disease: the yersiniabactin locus (*ybt*) was present in 163 (49%) of the BSI *K. pneumoniae*, with the site prevalence ranging from 19 to 67% (Fig. 4a); no significant difference in *ybt* prevalence was observed between S and SE Asia (Table 1). Nine of the 14 known chromosomally integrated *ybt* mobile-genetic elements (ICE*Kp*s [36, 75, 76]) and two *ybt* plasmids were detected. The most common were ICE*Kp*5 (*n* = 41), ICE*Kp*4 (*n* = 36) and ICE*Kp*10 (also encoding colibactin, *n* = 31), found across multiple study sites. The ICE*Kp*10-positive samples included 25 in clonal group 23 (ST23 plus related STs), wherein ICE*Kp*10 is a marker of the important globally distributed CG23-I sub-lineage [77].

The *iuc*, *iro*, *rmpA*, *rmpA2* and *peg-344* loci, typically carried on virulence plasmids, were commonly detected in our genome collection (28% *iuc*, 21% *iro*, 18% *rmpA* 16% *rmpA2*, 19% *peg-344*; 9% with all five). *Peg-344* and *iuc* have been suggested as the most predictive for hypervirulence [37]. We focus the remainder of our analyses on the *iuc* locus because its mechanism of action is well understood and its isogenic knockout mutants are clearly attenuated in mouse models of hypervirulent infection, unlike those for *peg-344* [32, 43–45].

The prevalence of *iuc* did not differ significantly between the S and SE Asian isolates, but *iuc* lineages and chromosomal STs of *iuc*-positive isolates were differentially distributed between the sampling sites (Fig. 4b, c, Tables 1 and 3). *Iuc* lineage 1 (*iuc1*, *n* = 66) was widely distributed (Fig. 4c) but more prevalent in SE Asia (27% vs 13% in S Asia, *p* = 0·011; Table 1). *Iuc1* is associated with the KpVP-1 virulence plasmid [78] and was detected among 15 *K. pneumoniae* STs, most commonly those known to be associated with hypervirulent infections: ST23 (*n* = 28), ST65 (*n* = 7) and ST86 (*n* = 7). *Iuc5* (*n* = 12) is associated with *E. coli* plasmids and was detected only in ST231 from India, while *iuc2* (associated with KpVP-2 [78]) was detected in a single ST380 isolate from Vietnam. *Iuc3* (*n* = 13) was detected in SE Asia (Cambodia, Vietnam, Laos) and Hong Kong (Fig. 4c), among eight distinct STs (Additional file 1: Table S1). We selected four *iuc3*-positive isolates from Vietnam and Laos for long-read sequencing and found each harboured a distinct and novel FIB_K_/FII *iuc3* plasmid (Table 4).

**Table 3:** Notable *iuc*-positive STs by region

Location	Total *iuc*+	Notable clone/s	*iuc* allele	*N iuc* (% of *iuc*/site)	ICE*Kp* (N)	*iro* allele (*N*)	*rmpA/2* (*N*)	ESBL/CP (*N*)
Hong Kong	6 (22%)	ST45	*iuc3*	1 (17%)	ICE*Kp*4 (1)	–	–	CTX-M-3 (1)
Vietnam	34 (43%)	* ST23	*iuc1*	13 (38%)	ICE*Kp*10^a^ (10) ICE*Kp*3 (1)	*iro1* (13)	(10)	CTX-M-14 (1)
		* ST25	*iuc3*	2 (6%)	ICE*Kp*1 (2)	*iro3* (2)	(2)	–
			*iuc1*	1 (3%)	–	*iro1* (1)	(1)	–
		* ST65	*iuc1*	2 (6%)	ICE*Kp*10 ^a^ (1)	*iro1* (2)	(2)	CTX-M-15, VEB-1 (1)
Thailand	0	–		–	–	–	–	–
Laos	23 (38%)	* ST23	*iuc1*	10 (44%)	ICE*Kp*10 ^a^ (10)	*iro1* (10)	(10)	CTX-M-63 (1)
		* ST65	*iuc1*	2 (6%)	ICE*Kp*10 ^a^ (2)	*iro1* (3)	(3)	–
		* ST86	*iuc1*	–	ICE*Kp*4 (1)	*iro1* (3)	(3)	–
		ST592	*iuc1*	10 (44%)	*–*	*iro1* (3)	(3)	–
Cambodia	4 (10%)	–	–	–	–	–	–	–
Nepal	3 (9%)	ST15	*iuc1*	2 (67%)	ICE*Kp*12 (2)	–	(2)	CTX-M-15 (2) OXA-232 (1)
India	24 (28%)	ST231	*iuc5*	12 (50%)	ICE*Kp5* (12)	–	–	CTX-M-15 (8) OXA-232 (8)
			*iuc* ukn	2 (8%)	ICE*Kp5* (2)	–	–	CTX-M-15 (2) OXA-232 (2)
		ST2096	*iuc1*	3 (13%)	ICE*Kp*5 (2)	*iro1* (1)	–	CTX-M-15 (2)
		* ST23	*iuc1*	2 (8%)	ICE*Kp*3 (1) ICE*Kp*10 ^a^ (1)	*iro1* (2)	(1)	CTX-M-15 (1)
		ST11	*iuc1*	1 (48%)	ICE*Kp*12 (1)	–	–	CTX-M-15, OXA-232 (1)

Total iuc+: total number of genomes carrying *iuc* per site (i.e. predicted as aerobactin-producing)

Notable clones: clones with ≥ 3 *iuc*-positive genomes at any single site, or carrying *iuc* in addition to ESBL and/or carbapenemase genes. *Known hypervirulent STs

*iuc* allele: *iuc* lineages predicted by *Kleborate.* unk = *iuc* lineage unknown

*N iuc*: number of genomes of each notable clone carrying *iuc* per site

ICEKp: ICEKp variants and the number of genomes of notable clones carrying these variants per site. ^a^ICE*Kp*10 carries *ybt* and *clb*

*iro* allele: *iro* lineage and the number of genomes of notable clones carrying these *iro* variants per site

*rmpA/2*: number of genomes of notable clones carrying the *rmpA* and/or *rmpA2* loci per site

ESBL/CP: ESBL and carbapenemase genes detected among notable clones and the number of genomes carrying these genes per site

**Table 4:** Novel virulence plasmids sequenced in this study and their host isolate properties

Isolate	Virulence plasmid (accession)	*iuc* lineage	AMR genes	AMR genes elsewhere in genome
Sample data and chromosomal typing				
	Size and *rep* type(s)			
2579 Nepal, 2016 ST15, KL112, ICE*Kp*12	p2579_1 (MK649822) 182,805 bp IncHI1B	*iuc1*	*–*	*qnrB1*, *strA^B*, *mphA*, *arr2*, *sulII^*, *dfrA14*, *blaOXA-232*, *blaCTX-M-15*, *blaTEM-30^*
BA6740 India, 2016 ST11, KL24, ICE*Kp*12	pBA6740_1 (MK649823) 226,590 bp IncFIB(pQIL), IncFIIK	*Iuc1*	*qnrB1*	*strA^B*, *rmtF*, *mphA*, *arr2*, *sulII*, *blaOXA-232*, *blaCTX-M-15*, *blaSHV-11*, *blaTEM-30^*
BA813 India, 2017 ST2096, KL64, ICE*Kp*5	pBA813_1 (MK649825) 273,676 bp IncFIB(Mar), IncFIB	*iuc1*	*bla*CTX-M-15, *bla*OXA-232, *bla*TEM-54^, *aadA2^*, *armA*, *msrE*, *mphE^*, *catA1^*, *sulI*, *tetD*, *dfrA12*, *dfrA14*	*Sat-2A*, *sulI*, *dfrA1*, *blaOXA-1*
BA6201 India, 2017 ST231, KL51, ICE*Kp*5	pBA6201_1 (MK649824) 195,016 bp IncFIA, IncFIB(pQIL), IncFIIK, IncFII	*iuc 1*	*rmtF*, *mphA*, *ermB^*, *catA1^*, *arr2*	*blaCTX-M-15*, *blaTEM-30^*
16114547 Laos, 2016 ST290, KL21	p16114547_1 (MK649829) 187,989 bp IncFIBK, IncFII	*iuc 3*	*qnrS1*, *tetA^*, *bla*TEM*-30^*	*blaSHV-26^*
1675474 Laos, 2015 ST7-1LV, KL54	p1675474_1 (MK649827) 181,647 bp IncFIBK, IncFII	*iuc 3*	*catA2^*, *sulII*	*qnrS1*, *tetA^*, *blaTEM-30^*
1675479 Laos, 2015 ST945-2LV, KL125	p1675479_1 (MK649828) 167,992 bp IncFIBK, IncFII	*iuc 3*	*strA^B*, *sulII*	*tetA*
130411–38618 Vietnam, 2011 ST17, KL127	p130411-38,618_1 (MK649826) 241,799 bp IncFIBK, IncFIIK	*iuc3*	*strA^B*, *aadA1*, *cmlA5*, *floR*, *arr2^*, *sulII*, *tetA*, *dfrA14*, *bla*OXA-10	

*rep types as per the PlasmidFinder v2.0 database are shown. All plasmids carried the *iuc* aerobactin synthesis operon. No plasmids carried the *iro* or *rmpA/rmpA2* virulence loci. Seven virulence plasmids also carried AMR (antimicrobial resistance) genes. Seven host isolates harboured additional AMR genes elsewhere in the genome. *ST* chromosomal multi-locus sequence type, *KL* K-locus. ^Inexact match

### Genotypic convergence of antimicrobial resistance and virulence

**Fig. 5:**
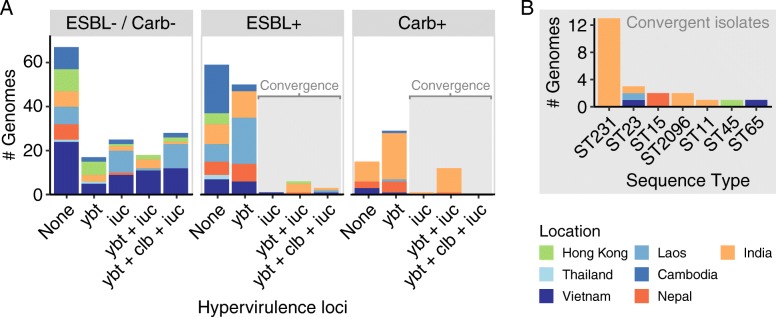
Convergence of virulence and antimicrobial resistance determinants. **a** Frequency of genomes carrying the yersiniabactin (*ybt*), colibactin (*clb*) and/or aerobactin (*iuc*) loci shown by ESBL and carbapenemase gene status. Bars are coloured by location as per legend. Grey shading indicates convergent isolates, i.e. those harbouring at least one ESBL and/or carbapenamase gene plus *iuc* with/without *ybt* and *clb*. **b** Chromosomal sequence types of convergent isolates. Bars are coloured by location as per legend

While strains carrying either AMR or hypervirulence determinants are of concern, those carrying both pose the greatest potential public health threat. The *ybt* virulence locus was significantly associated with ESBL *K. pneumoniae* (OR 1·6; 95% CI 1.06–2.55, *p* = 0·021) and CP *K. pneumoniae* (OR3·5; 95% CI 1.87–6.98, *p* < 0·0001). *Ybt* + ESBL *K. pneumoniae* BSI were detected at all sites (Fig. 5a) and exhibited a similar prevalence in both S and SE Asia (Table 1). *Iuc* was present in 13% of ESBL *K. pneumoniae* BSI (vs 43% among non-ESBL; OR 0·2; 95% CI 0.12–0.38, *p* < 0·0001) and 23% of CP *K. pneumoniae* (vs 30% among non-CP; OR 0·8; 95% CI 0.37–1.59, *p* = 0·6). Overall prevalence of *iuc* + ESBL *K. pneumoniae* was 6%. *Iuc* + ESBL isolates were detected at five of the seven sampling locations (Fig. 5a) and were more common in S than SE Asia (*p* < 0·001, Table 1). Conversely, *iuc* + CP *K. pneumoniae* BSI were detected only in India (*n* = 12 ST231, 14% of Indian isolates) and Nepal (*n* = 1 ST15, 3%).

Convergent AMR-hypervirulent isolates (organisms carrying *iuc* plus ESBL and/or carbapenemase genes) were seen across seven different STs circulating across this region, with a prevalence of 7.3% (Fig. 5b, Table 3). Long-read sequencing of four representative isolates of different STs revealed four novel mosaic plasmids. Three of these plasmids (and the four *iuc3* plasmids) harboured *iuc* plus AMR genes (1–10 AMR genes, encoding resistance to 10 drug classes; Table 4). Notably, one of these plasmids harboured *iuc1*, *bla*_CTX-M-15_, and eight additional AMR genes (plasmid pBA813_1 from isolate BA813, Table 4**).** While pBA813_1 was not predicted to encode the *tra* plasmid transfer machinery, the four *iuc3* plasmids and one of the *iuc1* plasmids (pBA6201_1, *iuc1* plus five AMR genes) contained a complete *tra* operon suggesting that they are capable of conjugative transfer. Consistently, we detected a high degree of sequence similarity between the *iuc3* plasmids carried by isolates of three distinct STs in Laos (p1675474 vs p16114547, > 99.9% nucleotide identity, > 89% coverage; p1675479 vs p1675474 and p1675479 vs p16114547, > 99% identity, > 59% coverage), supporting their dissemination within the local *K. pneumoniae* population or acquisition from a recent common source.

Comparison of chromosomal ST, *iuc* lineage, and AMR gene content indicates at least nine distinct AMR-virulence convergence events in our sample (Table 3). These include four distinct AMR element acquisitions by previously described hypervirulent STs (three in ST23 from Vietnam, Laos and India; one in ST65 from Vietnam), four distinct virulence plasmid acquisitions by previously described MDR STs (one in ST15 from Nepal; one in ST11 from India; one in ST2096 from India; one in ST231 from India; plus one in ST45 from Hong Kong). Notably, most of these organisms also harboured the additional virulence factors *ybt*, *iro* and *rmpA/rmpA2* (Table 3), supporting the interpretation that they are likely to manifest the hypervirulent phenotype. Three of the strains resulting from these AMR-virulence convergence events showed evidence of local clonal expansion: ST15 in Nepal (*n* = 2 isolates, 32 pairwise SNVs in an alignment of 5,709,381 bp, accounting for ≥ 99.5% of each genome), ST2096 in India (*n* = 2 isolates, 57 pairwise SNVs in an alignment of 5,489,606 bp, accounting for ≥ 96.2% of each genome) and ST231 in India (*n* = 13 isolates, 9 to 319 pairwise SNVs, mean 139.4 SNVs, mean alignment length 5,276,478 bp, accounting for mean 96.5% of each genome). The latter included two potential nosocomial transmission clusters (≤ 25 pairwise SNVs; *n* = 4 isolates carrying *iuc5* + *bla*_OXA-232_ with/without *bla*_CTX-M-15_; *n* = 2 isolates carrying *iuc5* + *bla*CTX-M-15, see Additional file 1: Table S1).

Each AMR-hypervirulent strain was detected only at one site, with no evidence of dissemination between countries. We did identify eight clusters of closely related *K. pneumoniae* strains (each separated by ≤ 100 SNVs, in a mean alignment length of 5,435,515 bp, accounting for mean 98.1% of each genome) that were detected in multiple countries, suggesting that regional spread of both MDR and hypervirulent *K. pneumoniae* does occur between Asian countries, as was recently described for CP *K. pneumoniae* in Europe [70]. However, while most of these clusters were either MDR or hypervirulent, none were both (Table 5). Five clusters were indicative of regional spread between SE Asian countries (ST101, ST17, ST65, ST86 and ST592); the other three clusters were detected in India plus either Laos (ST412), Hong Kong (ST14) or Nepal (ST15) (see Table 5 and Additional file 1: Table S1).

**Table 5:** Multi-country clusters of strains sharing a recent common ancestor

ST	Pairwise SNVs (mean)^a^	Locations	Sample ID	Year	ESBL/CP genes	ICEKp	Plasmid-borne virulence loci
ST17	33	Cambodia	COMRU-KPN-BC-2014-21	2014	–	Unk	–
		Vietnam	CM3425	2015	–	Unk	–
ST65	37–138^b^ (89)	Laos	1675485	2015	–	ICE*Kp*10^d^	*iro1*, *iuc1*, *rmpA*, *rmpA2*
		Laos	1675477	2015	–	ICE*Kp*10^d^	*iro1*, *iuc1*, *rmpA*, *rmpA2*
		Laos	16114200	2016	–	–	*iro1*, *iuc1*, *rmpA*, *rmpA2*
		Cambodia	COMRU-KPN-BC-2015-7	2015	–	ICE*Kp*10^d^	*iro1*, *iuc1*, *rmpA*, *rmpA2*
ST86	69	Vietnam	270210–16361	2010	–	ICE*Kp*4	*iro1*, *iuc1*, *rmpA*, *rmpA2*
		Laos	1623415	2016	–	ICE*Kp*4	*iro1*, *iuc1*, *rmpA*
ST101	73	Cambodia	KPN239	2014	CTX-M-15	–	–
		Laos	1690095	2015	CTX-M-15, CTX-M-27	–	–
ST592	70–91 (80)	Laos	1675487	2015	–		*iuc1*, *iro1*, *rmpA2*
		Laos	1675478	2015	–	–	*iuc1*, *iro1*, *rmpA2*
		Vietnam	CM3560	2015	–	–	*iuc1*, *iro1*, *rmpA2*
ST14	82	Hong Kong	V28	2016	–	–	–
		India	BA33569	2016	–	–	–
ST15	0–202 (84)^c^	Nepal	2427	2016	CTX-M-15, OXA-232, NDM-1	ICE*Kp*12	–
		Nepal	2455	2016	CTX-M-15, OXA-232, NDM-1	–	–
		Nepal	2703	2016	CTX-M-15, OXA-232, NDM-1	–	–
		Nepal	2725	2016	CTX-M-15, OXA-232, NDM-1	–	–
		Nepal	2557	2016	CTX-M-15	–	*iuc1*, *rmpA2*
		Nepal	2579	2016	CTX-M-15, OXA-232	–	*iuc1*, *rmpA2*
		India	BA12537	2017	CTX-M-15	ICE*Kp*12	–
ST412	86	Laos	16112578	2016	–	–	*iuc1*, *iro1*, *rmpA*, *rmpA2*
		India	BA2641	2017	–	–	*iuc1*, *iro1*, *rmpA*, *rmpA2*

Genomes were clustered at a threshold of ≤ 100 SNVs (i.e. all members of a cluster differ from at least one other member by fewer than 100 SNVs). Clusters comprising isolates from multiple countries are shown. *Unk* unknown. ^a^Pairwise SNV count; for clusters of size > 2 genomes the range and mean are shown. ^b^Cambodian isolate COMRU-KPN-BC-2015-7 differs from each of the Laos isolates by 67–96 SNVs. ^c^Indian isolate BA12537 differs from each of the Nepalese isolates by 81–202 SNVs. ^d^ICE*Kp*10 carries *ybt* and *clb*

## Discussion

This work represents the first broad genomic study of *K. pneumoniae* causing BSI in S/SE Asia and Hong Kong, regions that are facing a combination of community-acquired invasive hypervirulent *K. pneumoniae*, unregulated use of antimicrobials, and the emergence and spread of MDR pathogens*.*

The diversity of *K. pneumoniae* causing BSI in S/SE Asia represents a significant challenge to therapy using existing or novel agents. Our study revealed a highly diverse set of isolates, both in terms of chromosomal STs and surface polysaccharide loci. We were unable to stratify the isolates into hospital-associated and community-acquired cohorts, but anticipate that the former accounts for the majority of the diversity in our sample given that the majority of community-acquired *K. pneumoniae* BSIs are known to be caused by a small number of distinct clones with limited surface antigen diversity [38–40]. This is consistent with the hypothesis that the majority of hospital-associated BSIs originate from the patients’ personal gastrointestinal microbiota rather than from intra-hospital transmissions [67, 79]. The latter is highly relevant to the design of vaccines or other interventions targeting *K. pneumoniae* capsules or lipopolysaccharide, which are considered of high importance in response to increasing prevalence of ESBL and CP strains, both of which were common in our sampling locations (47% and 17% of all isolates, respectively). From our sampling strategy, we crudely estimate a non-cross-reactive capsule-targeted vaccine would need to include ≥ 16 serotypes in order to provide potential protection against > 50% of the BSI *K. pneumoniae* isolates across these study sites. The 16 most common K-loci would cover 61% of all ESBL and 68% of all CP *K. pneumoniae* across S/SE Asia. Alternatively, we estimate that a completely immunising vaccine targeting O1, O2 and O3b would hypothetically protect against 79% of the BSI *K. pneumoniae* isolates in this study, equating to 79% of all ESBL-containing isolates and 70% of CP.

Alongside the high prevalence of ESBL and carbapenemase genes, our data revealed high prevalence of known hypervirulence determinants with the *iuc* locus detected in 28% of all isolates included in this study, more than double the prevalence seen in previous studies focused outside of this region [78]. We suggest this likely reflects the combination of a comparatively greater prevalence of strains from community-acquired infections (particularly in SE Asia) plus comparatively elevated prevalence among MDR hospital-associated strains. Accordingly, 33 of 210 (16%) genomes predicted to be MDR also carried *iuc* (note includes 5 genomes that were MDR variants of known hypervirulent clones). While the *iuc* locus itself was common in both S and SE Asia, the distribution of *iuc* lineages differed (Table 1). Specifically, *iuc1* (associated with the characteristic KpVp-1 virulence plasmid [78]) and *iuc3* were more common in SE Asia where they were each detected among numerous distinct STs. This is consistent with their local dissemination or recent acquisition from a common source, a finding supported by the discovery of four novel but similar *iuc3* encoding plasmids in these isolates. Of greatest concern was the detection of at least nine distinct convergence events between AMR and hypervirulence (encompassing 7·3% of isolates) plus seven novel dual AMR + *iuc* containing plasmids, increasing the total number of convergent plasmids reported so far by almost 50% [49, 80–83]. Given that our isolate collection represented a snapshot from seven diagnostic laboratories, we predict that many additional convergent plasmid variants await future identification.

It remains unclear if convergent MDR-virulent *K. pneumoniae* plasmids and/or strains are fit for widespread dissemination or simply represent transient events that are rapidly purged from the population. The latter could explain the historical absence of reports of MDR hypervirulent infections and is consistent with the apparent lack of dissemination of any of the numerous reported convergent MDR ST23 variants, despite ample evidence that the susceptible hypervirulent ST23 clone is capable of global distribution [39, 40, 49, 84–87]. However, it is important to note that other factors have also been proposed; for example, the possibility that hypervirulent and MDR clones preferentially inhabit distinct ecological niches outside of the human host, thereby limiting the opportunity for transfer of genetic material between them; or that there may be physical and/or mechanistic barriers to genetic exchange [88]. Additionally, there is now clear evidence that at least a subset of the globally recognised MDR clones with acquired virulence plasmids are able to cause persistent local problems and spread more broadly: These include the CP ST231 *iuc +* strain reported here from the Indian hospital, a previously reported CP ST15 *iuc* + in a Pakistani hospital [53] and CP ST11 *iuc* + in China, which was able to disseminate for several years without detection [52, 57]. Fortunately, most MDR-virulent infections thus far appear to be hospital-associated rather than community-acquired, suggesting that unlike the hypervirulent clones, these convergent strains may be less able to spread outside of the hospital environment and/or unable to cause invasive disease in otherwise healthy hosts. Nevertheless, the combination of enhanced virulence plus limited treatment options is an important concern that warrants careful attention. Combined with our data indicating high prevalence and diversity of convergent strains in the S/SE Asian region, it is clear that there is a need for enhanced *K. pneumoniae* surveillance to rapidly identify and monitor convergent strains and/or plasmids.

A limitation of this work is that the data represents a retrospective convenience sample of *K. pneumoniae* BSI isolates by the participating diagnostic laboratories during routine activities during overlapping time periods. This prevented statistical comparisons between individual sites and should be considered when extrapolating gene prevalence information to the broader *K. pneumoniae* population. Additionally, we did not have access to patient clinical data, which has limited our interpretation of the data in the context of patient co-morbidities, and stratification of isolates into community-acquired and hospital-associated cohorts. We focussed on BSI, as this allowed for convenient and consistent retrospective identification of isolates associated with invasive disease for inclusion in the study. While this presentation arguably reflects the greatest clinical need, the siderophore virulence loci are known to be more prevalent in BSI compared to other infections [35], and hence the prevalence of *iuc* estimated from our sample may be higher than that of the broader population of *K. pneumoniae* causing infections in these regions. In addition, we note that our inferences about AMR are based on genotypic information, which is highly predictive of AMR in *K. pneumoniae* but not perfectly correlated [67, 89]. Nevertheless, our analyses reveal valuable insights and provide essential data to motivate enhanced public health surveillance.

## Conclusions

*K. pneumoniae* BSI isolates from South and Southeast Asia represent diverse STs, capsule and LPS serotypes, with high prevalence of MDR, hypervirulence-associated loci and convergent MDR-virulent strains. Our study represents a blueprint for genomic surveillance of emerging AMR pathogens in this region and we urge the coordination of similar activities internationally. By rapidly detecting resistance and virulence genes in the context of clonal and surface antigen diversity, our approach provides critical information that can be used to simultaneously track the emergence and dissemination of clinically important variants, guide antimicrobial therapy and assess potential mechanisms and targets for containment and intervention. In combination with rapid reporting and data sharing, this approach will permit researchers and public health professionals to recognise and control the growing public health threat of AMR *K. pneumoniae*.

## Additional files

Additional file 1: **Table S1.** Sample information and genotyping results for all BSI *Kp* included in this study.

Additional file 2: **Table S2.** Characteristics of non-*Kp* BSI isolate genomes. **Table S3.** K-locus prevalence among *K. pneumoniae* sensu stricto. **Table S4.** Predicted O type prevalence among *K. pneumoniae* sensu stricto.
