# Supplementary material for: Genomic surveillance for hypervirulence and multi-drug resistance in invasive Klebsiella pneumoniae from South and Southeast Asia
Source: Genome Med. 2020 Jan 16;12:11. doi: 10.1186/s13073-019-0706-y (PMC6966826; doi:10.1186/s13073-019-0706-y)
Supplement: Supplementary file 2 — Table S2. Characteristics of non-Kp BSI isolate genomes. Table S3. K-locus prevalence among K. pneumoniae sensu stricto. Table S4. Predicted O type prevalence among K. pneumoniae sensu stricto. [file 13073_2019_706_MOESM2_ESM.pdf]

SUPPLEMENTARY TABLES FOR:

Genomic surveillance for hypervirulence and multi-drug resistance in invasive *Klebsiella pneumoniae* from south and southeast Asia

Kelly L Wyres DPhil[1], To N T Nguyen[2], Margaret M C Lam PhD[1], Louise M Judd PhD[1], Nguyen van Vinh Chau MD[3], David A B Dance FRCPath[4,5,6], Margaret Ip MSc[7], Abhilasha Karkey MSc[5,8], Clare L Ling PhD[5,9], Thyl Miliya MD[10], Paul N Newton MRCP[4,5,6], Lan Nguyen[3], Amphone Sengduangphachanh [11], Paul Turner FRCPath[5,10], Balaji Veeraraghavan FRCP[12], Phat Voong Vinh MSc[2], Manivanh Vongsouvath[11], Nicholas R Thomson PhD[6,13], Stephen Baker PhD[2,14*] and Kathryn E Holt PhD[1,6]

[1] Department of Infectious Diseases, Central Clinical School, Monash University, Melbourne, Victoria 3004, Australia
[2] Oxford University Clinical Research Unit, Hospital of Tropical Diseases, Ho Chi Minh City, Vietnam
[3] The Hospital of Tropical Diseases, Ho Chi Minh City, Vietnam
[4] Lao-Oxford-Mahosot Hospital-Wellcome Trust Research Unit, Microbiology Laboratory, Mahosot Hospital, Vientiane, Laos.
[5] Centre for Tropical Medicine and Global Health, University of Oxford, Oxford, United Kingdom
[6] London School of Hygiene and Tropical Medicine, London, United Kingdom
[7] Department of Microbiology, The Chinese University of Hong Kong, Hong Kong Special Administrative Region, China
[8] Oxford University Clinical Research Unit, Patan Academy of Health Sciences, Kathmandu, Nepal
[9] Shoklo Malaria Research Unit, Mahidol-Oxford Tropical Medicine Research Unit, Faculty of Tropical Medicine, Mahidol University, Mae Sot 63110, Thailand.
[10] Cambodia Oxford Medical Research Unit, Angkor Hospital for Children, Siem Reap, Cambodia
[11] Lao-Oxford-Mahosot Hospital-Wellcome Trust Research Unit, Microbiology Laboratory, Mahosot Hospital, Vientiane, Lao PDR
[12] Department of Clinical Microbiology, Christian Medical College, Vellore, Tamil Nadu, India
[13] Wellcome Trust Sanger Institute, Hinxton, Cambridge, United Kingdom
[14] The Department of Medicine, The University of Cambridge, Cambridge, United Kingdom

* corresponding author:
Stephen Baker, Oxford University Clinical Research Unit, Hospital of Tropical Diseases, Ho Chi Minh City, Vietnam
Email; sbaker@oucru.org

Table S2: Characteristics of non-*Kp* BSI isolate genomes

	K. quasipneumoniae subsp. *quasipneumoniae*	*K. quasipneumoniae* subsp. *similipneumoniae*	*K. variicola*
**N**	5	20	9
**# STs**	4:<br>2 Vietnam<br>1 India<br>1 Nepal	17:<br>7 Cambodia<br>6 Vietnam<br>5 Laos	9:<br>3 Hong Kong<br>2 Cambodia<br>1 India<br>1 Laos<br>1 Nepal<br>1 Vietnam
**Common STs[a]**	ST1118-3LV (40%)	ST1191 (10%)<br>ST1124 (10%)<br>ST334 (10%)	-
**# K loci**	3	15	9
**Common K loci[a]**	KL107 (60%)	KL60 (15%)<br>KL10 (10%)<br>KL13 (10%)<br>KL103 (10%)	-
**# O types**	2	4	4
**Common O types[a]**	O3/O3a (80%)	O5 (55%)<br>O3/O3a (30%)<br>O12 (10%)	O3/O3a (44%)<br>O5 (33%)
**% ESBL+**	0	65%	0
**ESBL genes**	-	CTX-M-15 (35%)<br>CTX-M-27 (10%)<br>SHV-2a (10%)<br>CTX-M-14 (5%)<br>CTX-M-9 (5%)	-
**% Carb+**	20%	0	0
**Virulence determinants**	-	ICE*Kp*3 + *iuc1* + *iro1* + *rmpA* + *peg-344*<br>(n=1 ST367 from Vietnam)	*ybt* plasmid lineage<br>(n=1 ST209-1LV from Cambodia)

[a] Only O types detected in >1 genome are shown.

Table S3: K locus prevalence among *K. pneumoniae sensu stricto.*

	K locus	Prevalence (%)								
		Cambodia	Hong Kong	India	Laos	Nepal	Vietnam	Mean	ESBL+	Carb+
1	KL2	12	11	2	11	6	10	9	3	0
2	KL1	2	7	2	16	0	19	8	2	0
3	KL24	5	7	1	23	0	1	6	11	2
4	KL51	0	0	22	2	3	3	5	11	23
5	KL62	7	11	5	0	0	5	5	4	4
6	KL112	0	0	2	0	22	0	4	6	11
7	KL57	0	7	2	7	0	6	4	1	0
8	KL10	5	0	1	7	3	5	3	6	0
9	KL23	7	4	0	2	3	4	3	2	0
10	KL13	2	0	0	0	16	0	3	3	0
11	KL64	0	0	18	0	0	0	3	3	21
12	KL102	7	4	0	3	0	3	3	2	0
13	KL117	0	7	4	0	3	0	2	2	4
14	KL54	2	7	1	2	0	1	2	1	0
15	KL122	10	0	0	0	0	1	2	3	0
16	KL105	0	0	0	0	9	1	2	2	5
17	KL3	0	4	0	2	3	1	2	3	0
18	KL149	0	4	1	0	0	3	1	1	2
19	KL19	0	0	4	0	0	4	1	3	2
20	KL52	2	0	0	2	3	0	1	1	0
21	KL39	0	4	0	0	3	0	1	1	0
22	KL7	5	0	0	2	0	0	1	1	0
23	KL17	2	0	2	2	0	0	1	1	4
24	KL5	0	4	0	0	0	3	1	0	0
25	KL25	5	0	0	0	0	1	1	1	0
26	KL28	5	0	0	0	0	1	1	2	0
27	KL27	2	0	0	0	3	0	1	1	0
28	KL125	0	4	0	2	0	0	1	0	0
29	KL21	2	0	0	2	0	1	1	1	0
30	KL103	0	4	0	0	0	1	1	1	0
31	KL12	0	4	0	0	0	1	1	1	0
32	KL20	0	0	1	0	0	4	1	0	0
33	KL9	0	4	1	0	0	0	1	0	0
34	KL107	0	0	0	0	3	1	1	1	0
35	KL45	0	0	0	2	0	3	1	1	2
36	KL15	2	0	0	2	0	0	1	2	0
37	KL114	0	4	0	0	0	0	1	0	0
38	KL106	0	0	0	3	0	0	1	1	0
39	KL148	0	0	0	3	0	0	1	1	0
40	KL162	0	0	0	0	0	3	0	0	0
41	KL163	0	0	0	0	0	3	0	1	2
42	KL31	0	0	1	0	0	1	0	0	2
43	KL34	2	0	0	0	0	0	0	0	0
44	KL111	2	0	0	0	0	0	0	0	0
45	KL135	2	0	0	0	0	0	0	1	0
46	KL157	2	0	0	0	0	0	0	1	0
47	KL35	2	0	0	0	0	0	0	0	0
48	KL8	2	0	0	0	0	0	0	1	0
49	KL128	0	0	0	2	0	0	0	0	0
50	KL136	0	0	0	2	0	0	0	1	0
51	KL30	0	0	0	2	0	0	0	1	2
52	KL56	0	0	0	2	0	0	0	0	0
53	KL63	0	0	0	2	0	0	0	0	0
54	KL142	0	0	0	0	0	1	0	1	0
55	KL127	0	0	0	0	0	1	0	0	0

56	KL164	0	0	0	0	0	1	0	0	0
57	KL22	0	0	0	0	0	1	0	0	0
58	KL42	0	0	0	0	0	1	0	0	0
59	KL81	0	0	0	0	0	1	0	0	0
60	KL124	0	0	1	0	0	0	0	1	0
61	KL33	0	0	1	0	0	0	0	0	0
62	KL43	0	0	1	0	0	0	0	0	0
63	KL50	0	0	1	0	0	0	0	1	0
	Unk	0	0	24	0	19	4	8	11	18

K-loci are ordered by highest to lowest mean prevalence across all sites. Note that the Thai site is excluded from these calculations due to small sample size (n=4). Unk; unknown.

Table S4: Predicted O type prevalence among *K. pneumoniae sensu stricto*.

O type	Prevalence (%)								
	Cambodia	Hong Kong	India	Laos	Nepal	Vietnam	Mean	ESBL+	Carb+
O1	67	48	35	69	66	50	56	55	44
O2	12	26	15	8	16	15	15	13	16
O1/O2[a]	0	4	11	0	0	3	3	5	7
O3b	10	11	6	10	3	9	8	6	4
O4	2	4	0	2	3	4	2	3	0
O5	7	4	0	0	0	4	2	1	0
O3/O3a	0	0	0	8	0	3	2	3	0
OL101	2	0	0	0	6	1	2	1	0
OL103	0	4	0	2	0	1	1	1	0
OL104	0	0	2	0	0	0	0	1	2
OL102	0	0	0	0	0	1	0	0	0
Unk	0	0	31	2	6	10	8	12	28

Predicted O types are ordered by highest to lowest mean prevalence across all sites. Note that the Thai site is excluded from these calculations due to small sample size (n=4).

[a] It was not possible to confidently distinguish between O1 and O2 for a minority of genomes (n=12 in total). The prevalence of these genomes is shown here by site and among ESBL+/Carb+ isolates. These values are added at the second position in the cumulative prevalence plot (**Figure 2B**). Unk; unknown
